# Mannich Base as Corrosion Inhibitors for N80 Steel in a CO_2_ Saturated Solution Containing 3 wt % NaCl

**DOI:** 10.3390/ma12030449

**Published:** 2019-02-01

**Authors:** Mingjin Tang, Jianbo Li, Zhida Li, Luoping Fu, Bo Zeng, Jie Lv

**Affiliations:** 1 School of Chemistry and Chemical Engineering, Southwest Petroleum University, Chengdu 610500, China; fuluoping@outlook.com (L.F.); zengbo_2014@icloud.com (B.Z.); jlv6@me.com (J.L.); 2 Faculty of Engineering, Computer & Mathematical Sciences, The University of Adelaide, Adelaide, SA 5005, Australia; lzd038@163.com

**Keywords:** corrosion inhibitor, electrochemical, AFM, CO_2_ corrosion

## Abstract

In this paper, a corrosion inhibitor containing nitrogen atoms and a conjugated π bond was synthesised, and its final product synthesised by the optimal conditions of the orthogonal test results is named multi-mannich base (MBT). The corrosion inhibition effect on the N80 steel sheet of the corrosion inhibitor was evaluated in a CO_2_ saturated solution containing 3 wt % NaCl; the corrosion rate was 0.0446 mm/a and the corrosion inhibition rate was 90.4%. Through electrochemical and adsorption theory study, MBT is a mixed corrosion inhibitor that mainly shows cathode suppression capacity. The adsorption of MBT on the surface of the steel sheet follows the Langmuir adsorption isotherm; it can be spontaneously adsorbed on the surface of the N80 steel sheet, which has a good corrosion inhibition effect. The surface of the N80 steel sheet was microscopically characterised by atomic force microscope (AFM). It can be seen from the results that the N80 steel sheet with MBT added is significantly different from the blank control group; the surface of the steel sheet is relatively smooth, indicating that MBT forms an effective protective film on the surface of N80 steel, which inhibits the steel sheet.

## 1. Introduction

To improve oil and gas recovery, CO_2_ injection is used in the exploitation of oil, natural gas, coalbed methane and shale gas [1]. However, CO_2_ injection is accompanied by CO_2_ corrosion [2]. Corrosion will decrease the mining yield, resulting in oil wells failure, shutting down and even cause safety accidents, which will have a serious impact on the development of the oilfield and the economic benefits of the market [3,4,5,6]. The most common and effective way to control corrosion is to use chemical additives that reduce corrosion at very small dosages [7,8]. In recent years, various corrosion inhibitors have been successfully applied to the industry, and the corrosion has been controlled obviously and effectively [9,10].

So far, phosphorus and nitrogen-containing compounds have been widely used as corrosion inhibitors, including organic phosphorus, nitrogen-containing compounds and phosphorus-containing organic water-soluble polymers [11]. Phosphorus-containing corrosion inhibitors have excellent corrosion inhibition properties. However, they have some fatal defects; for example, phosphorus compounds are difficult to degrade in water and usually lead to eutrophication. The widespread use of this chemical may cause oxygen deficiency in the water, which leads to the death of aquatic organisms [12]. Therefore, in the last few years, the industry has not only required the effectiveness of chemical compounds but also their safety [13]. Today, chemical emissions are strictly controlled through legislation. Therefore, finding an alternative solution, namely the effective control of corrosion by green corrosion inhibitors, is the most important research direction at the moment. Chemical products identified as “green” are based on criteria: non-toxic and degradable [14,15].

In this paper, salicylaldehyde, diethylenetriamine, formaldehyde and acetone were selected to synthesise corrosion inhibitor MBT containing nitrogen atoms with lone pairs and a conjugated π bond. The structure was characterised by infrared radiation spectroscopy (IR) and nuclear magnetic resonance spectroscopy (^1^H NMR). The product was evaluated for corrosion inhibition by a weight loss method. Finally, the mechanism of corrosion inhibition was studied by atomic force microscopy (AFM) and electrochemical methods.

## 2. Experiment

### 2.1. Main Experimental Materials and Instruments

Salicylaldehyde C_7_H_6_O_2_, diethylenetriamine C_4_H_13_N_3_, ethanol C_2_H_6_O, acetone C_3_H_6_O, formaldehyde HCHO: AR, Chengdu Kelong Chemical Reagent Factory, Chengdu, China.

WQF-520 infrared spectrometer: Beijing Ruili Analytical Instrument (Group) Co. Ltd., Beijing, China; Bruker AVANCE III HD 400 nuclear magnetic resonance spectrometer: Bruker Corporation, Billerica, MA, USA; IVICMSTAT electrochemical workstation: Ivium Technologies, Eindhoven, The Netherlands; SPM-9600 atomic force microscope: Shimadzu Corporation, Kyoto, Japan.

### 2.2. Synthesis of Corrosion Inhibitor

First, in a three-necked flask equipped with a condenser and a thermometer, 20 mL of ethanol was added as a solvent. When the reactant reached a certain temperature, a certain amount of salicylaldehyde and diethylenetriamine were added in a certain ratio. After a period of reaction, the reaction was terminated to give the product intermediate mannich base (MB-1).

**Scheme 1:**
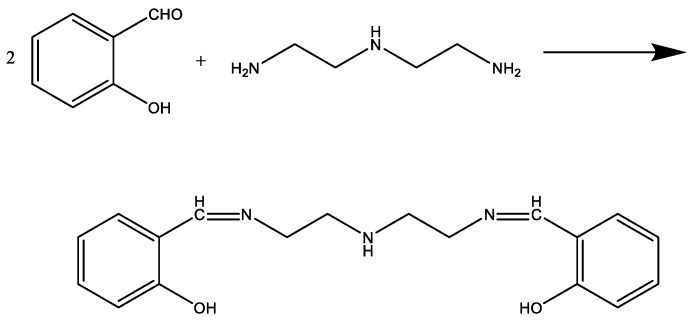
The synthesis of MB-1.

**Scheme 2:**
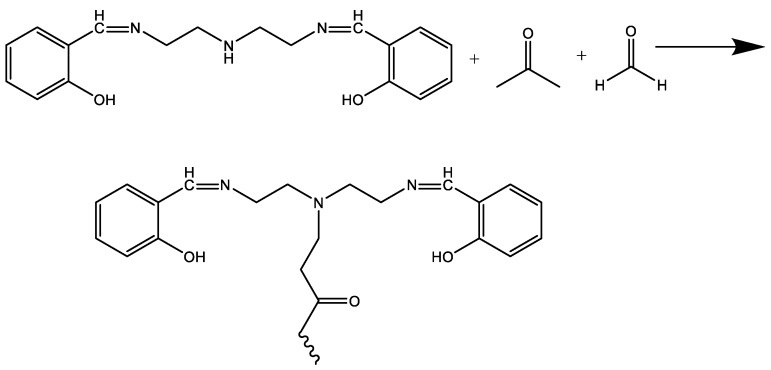
The synthesis of MB-2.

Then, a certain amount of acetone and formaldehyde were added in order, being kept at a constant reaction temperature for a certain period, and then the reaction was terminated to obtain the target product mannich base (MB-2). The synthesis of MB-1 and MB-2 is shown in Scheme 1 and Scheme 2.

### 2.3. Weight Loss Measurements

A hanging N80 steel test piece (50.00 mm × 13.00 mm × 1.50 mm: length, width, height) was ground with sand paper to give a surface with a uniform metal luster [16]. To calculate the superficial area of the test piece, the carbon steel was deoiled with putty powder and then immersed in ethanol for 5 min, dried with an air blower, wrapped in filter paper, maintained in a dryer and weighed to 0.0001 g.

Then the test piece was immersed in a bottle containing 500 mL of CO_2_ saturated solution and 3 wt % NaCl saline water with and without inhibitors at 70 °C for 72 h. According to the weight loss of each test piece and Equations (1) and (2), the rate of corrosion of the test piece and efficiency of the corrosion inhibitor at different concentrations could be calculated [4].

(1) $$v=\frac{8.76\times 10^{4}\times \Delta m}{\rho At},$$

where *ν*, Δ*m*, *ρ*, *A* and *t* are the corrosion rate (mm/a), weight loss (g), test sample density (g/cm^3^), exposed sample area (cm^2^) and immersion time (h), respectively. The inhibition efficiency (*η*) of the inhibitor was calculated by Equation (2):

(2) $$\eta \left(\%\right)=\left(1-\frac{v}{v^{0}}\right)\times 100,$$

where *ν* and *ν*^0^ are the corrosion rates calculated from the weight loss with and without the inhibitor.

### 2.4. Electrochemical Measurements

The potentiodynamic polarisation curve method was used to measure the Tafel polarisation curve. When the self-corrosion potential (Ec) of the system was stable, the cathode and anode scanning was from 250 mV to 300 mV. The polarisation curves of the N80 steel sheets with different concentrations of corrosion inhibitors were measured [4]. According to the relevant theory of electrochemistry, the corrosion inhibition rate (*η*) of the corrosion process can be calculated by Equation (3) [17]:

(3) $$\eta =\frac{i^{0}-i}{i^{0}}\times 100,$$

where *i*^0^ and *i* are the corrosion current densities without and with the inhibitor, respectively.

The electrochemical impedance spectroscopy (EIS, Ivium Technologies, Eindhoven, The Netherlands) experiments were performed by applying a sinusoidal voltage signal of 10 mV; the frequency range was 0.01–10^−5^ Hz; and the alternating current excitation signal amplitude was 10 mV. Impedance diagrams of the N80 steel sheets with different concentrations of corrosion inhibitors were measured. The inhibition efficiency (*η*) was determined from the EIS data using Equation (4):

(4) $$\eta =\frac{R_{t}-R_{t}^{0}}{R_{t}}\times 100,$$

where *R*_t_ and *R*_t_^0^ are the charge transfer resistance in the absence and presence of inhibitors, respectively.

### 2.5. AFM Analysis

Atomic force microscopy (AFM, Shimadzu Corporation, Kyoto, Japan) was used for characterizing the surface morphology and measuring surface roughness in a CO_2_ saturated solution containing 3 wt % NaCl without and with corrosion inhibitors. The two- and three-dimensional AFM images of steel specimens were taken in the range from 0 to 5 μm at room temperature [18].

## 3. Results and Discussion

### 3.1. IR Characterisation Results

**Figure 1:**
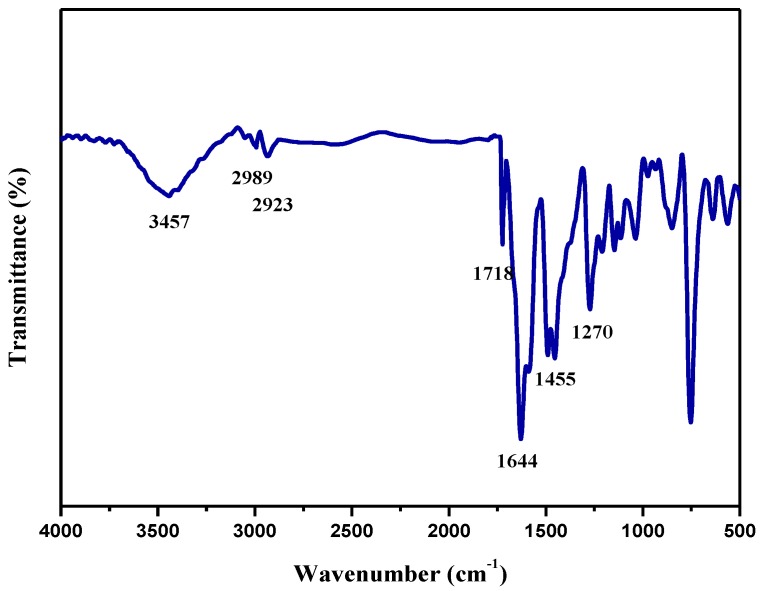
IR spectrum of MB-2.

The IR spectrum of MB-2 is shown in Figure 1. It is demonstrated that the stretching vibration peak attributed to the carbonyl group in salicylaldehyde does not appear at 1740 cm^−1^ to 1720 cm^−1^. The stretching vibration peak attributed to the C=N bond at 1644 cm^−1^ indicates that the salicylaldehyde reacts with diethylenetriamine to form a compound containing C=N and the aldehyde group disappears. The absorption peak at 3457 cm^−1^ and 1270 cm^−1^ is attributed to the stretching vibration of the O–H bond and C–O bond of phenol; the absorption peak at 2989 cm^−1^ and 2923 cm^−1^ is attributed to the stretching vibration of methyl (CH_3_) and methylene (CH_2_); the bending vibration of the N–H bond belonging to the secondary amine did not occur at 1580 cm^−1^ to 1490 cm^−1^, indicating that the hydrogen on the secondary amine reacted with acetone and formaldehyde, and the secondary amine disappeared into a tertiary amine; the absorption peak at 1718 cm^−1^ is attributed to the stretching vibration of C=O. IR characterization results showed that the synthesized MB-2 was the target product.

### 3.2. Optimization of MB-2 Synthesis Conditions

Through orthogonal experiments, the three-factor four-level L_16_(4^3^) orthogonal table was selected to investigate the influencing factors of MB-2 synthesis, including the ratio of reactants, reaction time and reaction temperature. Considering that both methyl groups of acetone react, the amount of acetone is fixed to adjust the ratio of MB-2 to formaldehyde. According to the product synthesised by the orthogonal experiment, when the amount was 200 mg/L, the corrosion inhibition performance was evaluated in a CO_2_ saturated solution containing 3 wt % NaCl.

**Scheme 3:**
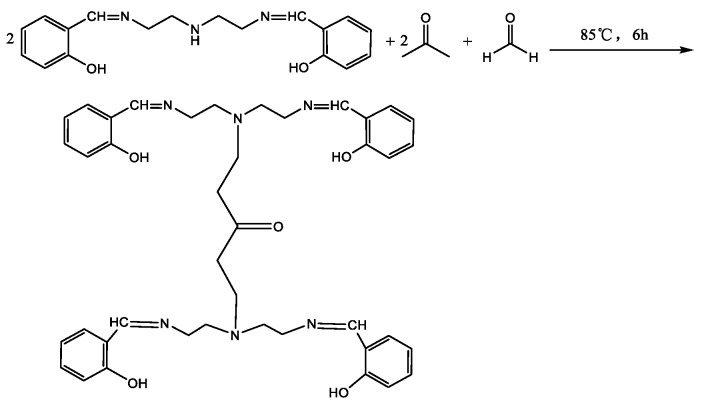
The synthesis of MBT.

The experimental factors and levels are shown in Table 1. The results of orthogonal experiments are shown in Table 2. It can be seen from Table 2 that the optimal reaction conditions for the synthesis of MB-2 are: reaction temperature 85 °C; reactant ration(MB-2):n(formaldehyde):n(acetone) = 2:2:1; and reaction time 6 h. The final product synthesised by the optimal conditions of the orthogonal test results is named MBT, and the reaction equation for the synthesis is shown in Scheme 3.

### 3.3. ^1^H NMR Analysis

**Figure 2:**
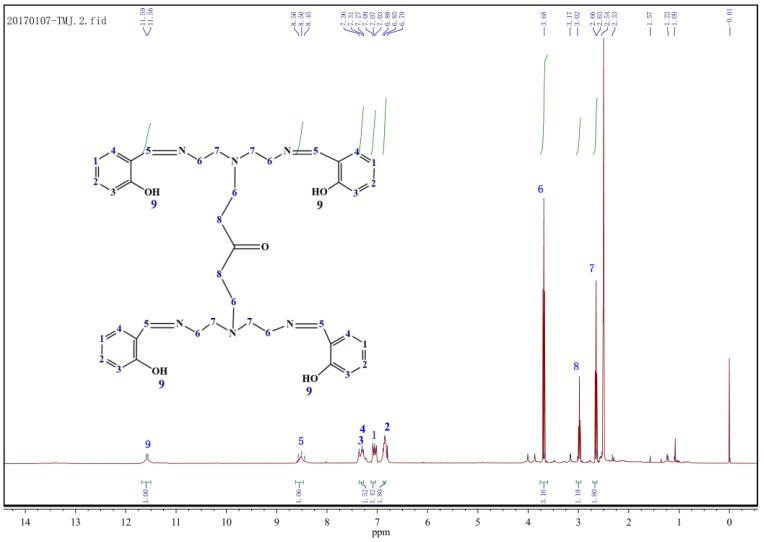
^1^H NMR spectrum of MBT.

The ^1^H NMR spectrum of MBT is shown in Figure 2. It can be seen from Figure 2 that the absorption peak at chemical shift *δ* = 12.00~11.00 is attributed to –OH; the absorption peak at *δ* = 8.56~8.45 is attributed to –CH=N; the absorption peak at *δ* = 7.36~6.79 is attributed to benzene (–CH); *δ* = 3.68~3.65, 3.02, the absorption peak at 2.63 is attributed to –CH_2_ at different positions; and the absorption peak at *δ* = 2.5 belongs to the solvent peak of Dimethyl sulfoxide (DMSO) and is characterized by IR. Combined with IR characterisation, the results indicate that MBT is the target product of synthesis.

### 3.4. Concentration Effect of MBT on Corrosion Inhibition

**Figure 3:**
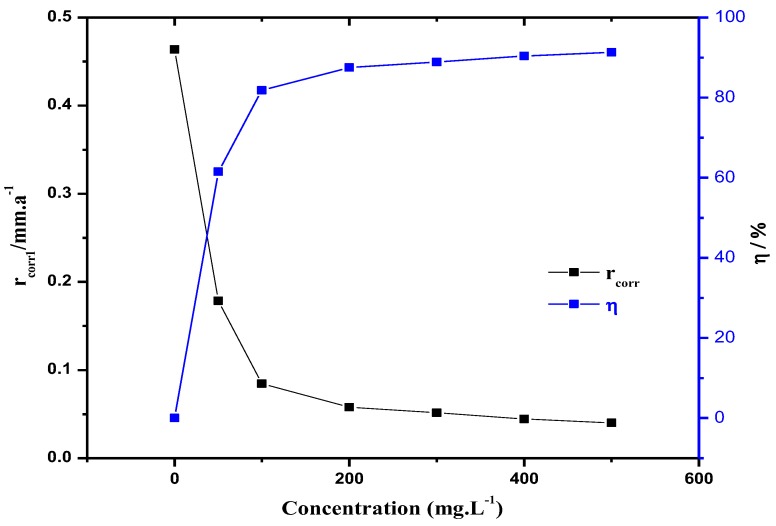
Effect of the dosage of MBT on the corrosion rate and corrosion inhibition rate of the N80 steel sheet.

The corrosion inhibition performance of the corrosion inhibitor MBT was evaluated by the static weight-loss method. Test conditions: temperature 70 °C, pH = 5.6, ρ(NaCl) = 30 g/L, and constant temperature water bath 72 h. The corrosion inhibition effect of MBT on a N80 steel sheet in a CO_2_ saturated solution containing 3 wt % NaCl was investigated under different dosages. The result is shown in Figure 3.

According to Figure 3, in the case of different dosages of MBT (0, 50, 100, 200, 300, 400, 500 mg/L), when the amount of MBT was 200 mg/L, the N80 steel sheet corrosion rate was 0.0579 mm/a. The corrosion rate of the steel sheet decreases with the increase of the corrosion inhibitor concentration, indicating that MBT can form an effective protective film on the metal surface. When the amount of corrosion inhibitor is 400 mg/L, the corrosion rate is 0.0446 mm/a, and the corrosion inhibition rate can reach 90.4%.

### 3.5. Temperature Effect of MBT on Corrosion Inhibition

In order to study the corrosion inhibitor for a CO_2_ saturated solution containing 3 wt % NaCl, the corrosion inhibition effect at different temperatures (40, 50, 60, 70, 80, 90 °C), the dosage of corrosion inhibitor in this experiment is 400 mg/L, and the evaluation time 72 h.

**Figure 4:**
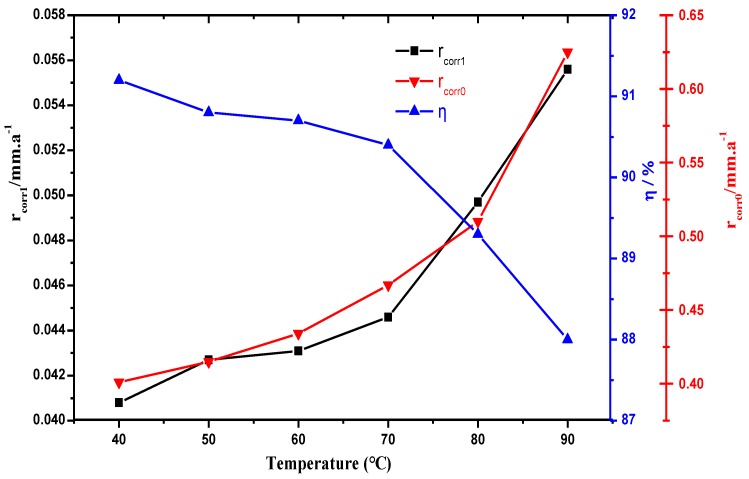
Evaluation of temperature on corrosion rate and corrosion inhibition rate.

As shown in Figure 4, the increasing temperature increases the corrosion rate in the absence (r_coor0_) and appearance of MBT solution (r_coor1_). This is because as the temperature increases, the adsorption capacity of the corrosion inhibitor decreases, the desorption capacity increases, and the corrosion rate of the steel sheet itself increases as the temperature is higher.

### 3.6. Discussion on Corrosion Inhibition Mechanism

#### 3.6.1. Polarization Curve Data Analysis

**Figure 5:**
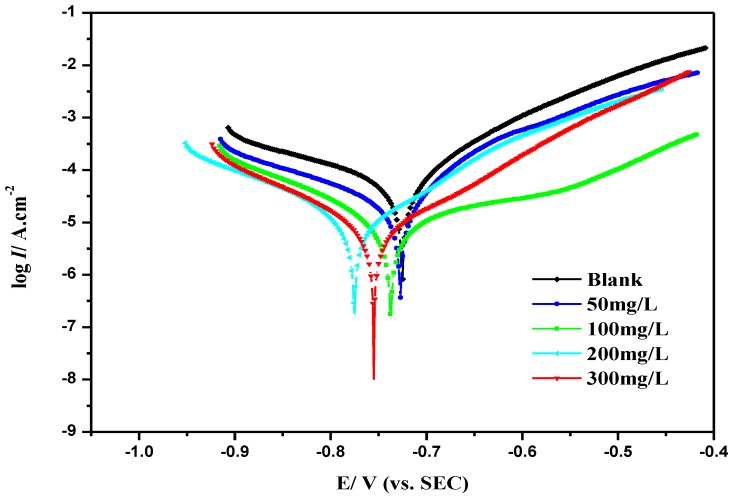
Tafel curve of N80 steel with different concentrations of MBT.

The inhibition mechanism of MBT was studied by the Tafel polarization curve method. Different concentrations (0 mg/L, 50 mg/L, 100 mg/L, 200 mg/L, 300 mg/L) of MBT were added, and the corrosive medium was a CO_2_ saturated solution containing 3 wt % NaCl at a temperature of 25 °C. The Tafel curve is shown in Figure 5.

It is shown in Figure 5 that when the different concentrations of MBT are added, the polarization curve moves downward as a whole, that is, the corrosion current density decreases. As the concentration of the corrosion inhibitor increases, the corrosion current density decreases. Since the corrosion current density is proportional to the corrosion rate, the concentration of the MBT increase made the corrosion rate drop, which is consistent with the results of the weight loss experiment. It can be seen from Figure 5, that after the addition of MBT, the polarization curves of the cathode and anode move in the direction of low corrosion current density, so MBT reacts to both the cathode and anode.

The corrosion potential (Ecorr), corrosion current density (Icorr), Tafel slope (βa and βc) and corrosion inhibition rate (*η*) calculated by the equation are shown in Table 3. It can be seen in the table that the corrosion inhibitor MBT moves the self-corrosion potential of the N80 steel sheet negatively; it shows that the corrosion inhibitor blocks the cathode process, and the self-corrosion potential moves in the negative direction (fluctuating around 30 mV) [19,20], so MBT is a mixed type corrosion inhibitor mainly for suppressing the cathode.

#### 3.6.2. Electrochemical Impedance Spectroscopy Data Analysis

**Figure 6:**
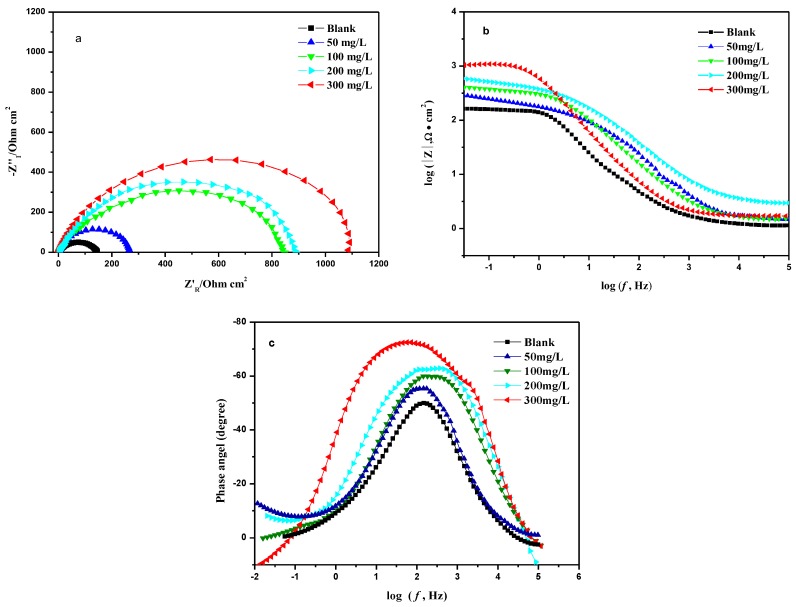
Nyquist (**a**), Bode-modulus (**b**) and Bode-phase angle (**c**) diagrams of N80 steel sheets with different concentrations of MBT.

In order to further study the corrosion inhibition mechanism of MBT on steel sheets, the impedance of the system was measured by an AC impedance method. Figure 6 is an impedance spectrum of MBT with different concentrations added.

**Figure 7:**
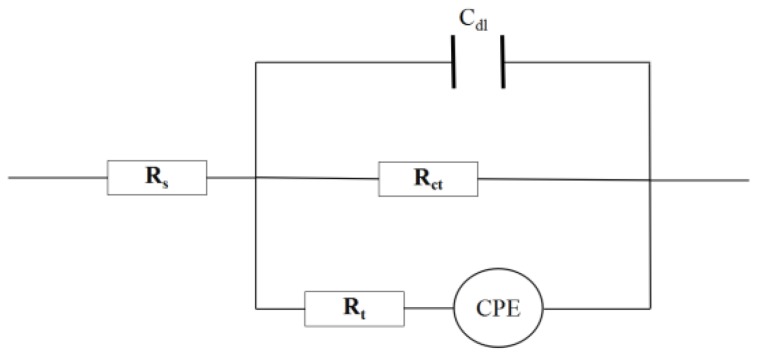
Fitting equivalent circuit diagram.

It can be seen from Figure 6 that the Nyquist diagram of the blank sample and the added corrosion inhibitor is a set of capacitive reactance arcs with a semicircular shape; however, the impedance of the system changes significantly after adding different concentrations of MBT compared with the blank. In addition, the capacitive anti-arc increases with the increase of corrosion inhibitor concentration, and the Bode-modulus value and the phase angle Bode-phase angle increase obviously, indicating that the corrosion inhibitor can be adsorbed on the surface of the N80 steel sheet to increase the transmission resistance to corrosion of the steel sheet, thereby reducing the corrosion rate of the metal. The EIS diagram was fitted using ZSimpWin software (ZSimDemo3.30d), and the equivalent circuit was obtained as R(C(R(QR))), as shown in Figure 7.

In Figure 7, R_S_ represents the solution resistance; R_ct_ represents the transfer resistance of the N80 steel sheet corrosion reaction charge; R_t_ and CPE represent the metal interface film resistance and constant phase element (CPE) to replace a double layer capacitance with a more accurate fit [21], the impedance of CPE was calculated following Equation (5); and C_dl_ represents the capacitance. The fitting parameters are shown in Table 4.

(5) $$Z_{\mathrm{CPE}}=\frac{1}{Y_{0}jw^{n}},$$

where Y_0_ is CPE constant; *j* is the imaginary number; *w* is the angular frequency; and *n* is the phase shift (which represents the deviation from ideal behavior).

From the data analysis in Table 4, as the intrusion time of N80 steel increases, the charge transfer resistance R_ct_ and film resistance R_t_ in the different corrosion inhibitor concentration solutions increase with the concentration of the corrosion inhibitor; the compactness of R_t_ and MBT adsorption film formation is related to the thickness, which indirectly indicates that the MBT molecule is effectively adsorbed by the N80 steel sheet. The solution resistance R_s_ does not change much and is significantly smaller than the film resistance R_t_ and charge transfer resistance R_ct_. Combined with the analysis of the Nyquist diagram, it is fully demonstrated that the corrosion inhibition effect is mainly absorbed by the corrosion inhibitor molecules on the metal surface, which changes the interface properties of the N80 steel surface and the electric double layer structure, thus achieving a good corrosion inhibition effect [22]. The calculated corrosion inhibition effect is basically consistent with the results obtained by the above weight loss experiment and the polarization curve method.

### 3.7. MBT Adsorption Mode

The MBT molecule is adsorbed on the surface of the metal to form a dense adsorption film, which acts as a corrosion inhibitor, and the stronger the adsorption capacity of the molecule on the metal surface, the better the corrosion inhibition effect [23]. The adsorption of MBT molecules on the metal surface is based on the structure, spatial distribution of the groups in the MBT molecule and the surface morphology of the metal; the adsorption modes are Temkin, Langmuir, Frumkin and Freundkick.

**Figure 8:**
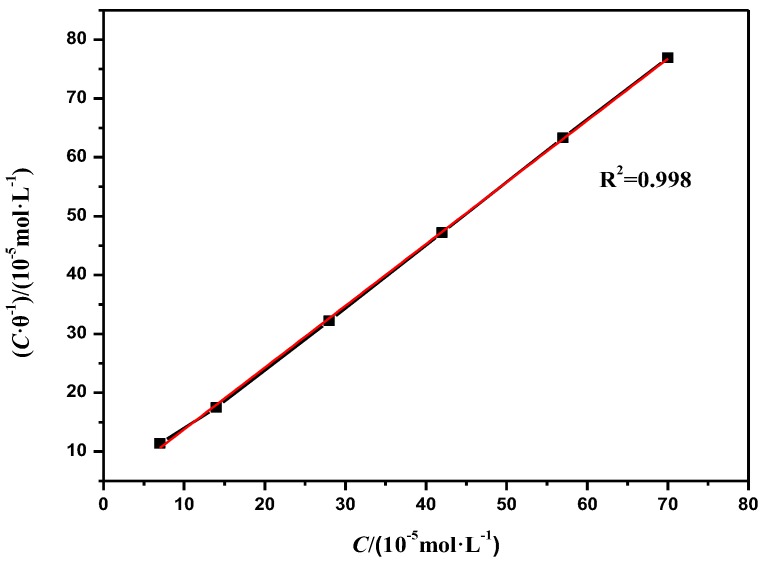
Langmuir isothermal adsorption curve of corrosion inhibitor MBT.

The above four adsorption isotherms were used to fit the data obtained by the polarisation curve method and the impedance method. It was found to be in accordance with the Langmuir adsorption equation, and the Langmuir adsorption curve is shown in Figure 8.

From Figure 8, it can be concluded that K_ads_ = 1.701 × 104 L/mol and R^2^ = 0.998, indicating that C/θ has a relatively good linear relationship with concentration C. The results show that at 70 °C, in a CO_2_ saturated solution containing 3 wt % NaCl, the adsorption of MBT on the surface of the N80 steel sheet follows the Langmuir isotherm adsorption. The formula for the adsorption free energy $\Delta G_{\mathrm{ads}}$ of the corrosion inhibitor MBT is as follows:

(6) $$\Delta G_{\mathrm{ads}}=-\mathrm{RTln}\left(55.5K_{\mathrm{ads}}\right),$$

Calculate $\Delta G_{\mathrm{ads}}=-39.25\mathrm{KJ}/\mathrm{mol}$ < 0 according to the adsorption equilibrium constant K_ads_ and Equation (6), indicating that the corrosion inhibitor MBT is capable of spontaneous chemical adsorption on the N80 steel sheet, which has a good corrosion inhibition effect.

### 3.8. Corrosion Inhibition Surface Morphology Analysis

**Figure 9:**
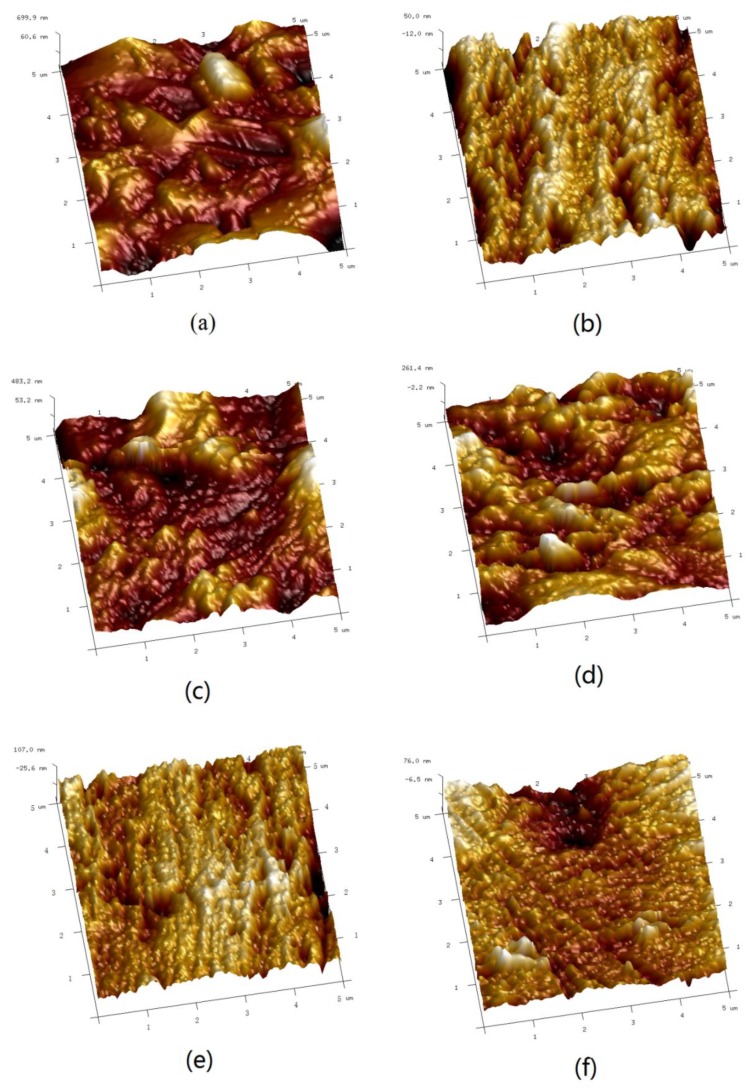
AFM three-dimensional image of N80 steel sheet: (**a**) blank sample; (**b**) initial uncorroded sample; (**c**–**f**) added 50 mg/L, 100 mg /L, 200 mg/L, 300 mg/L MBT.

In order to study the corrosion inhibition ability of the corrosion inhibitor MBT, the surface of the N80 steel sheet was characterised by AFM. The N80 steel sheet was immersed with different concentrations of corrosion inhibitor MBT in a CO_2_ saturated solution containing 3 wt % NaCl, and the water bath was kept at 70 °C for 72 h. The three-dimensional and planar topography of the N80 steel sheet surface is shown in Figure 9.

**Figure 10:**
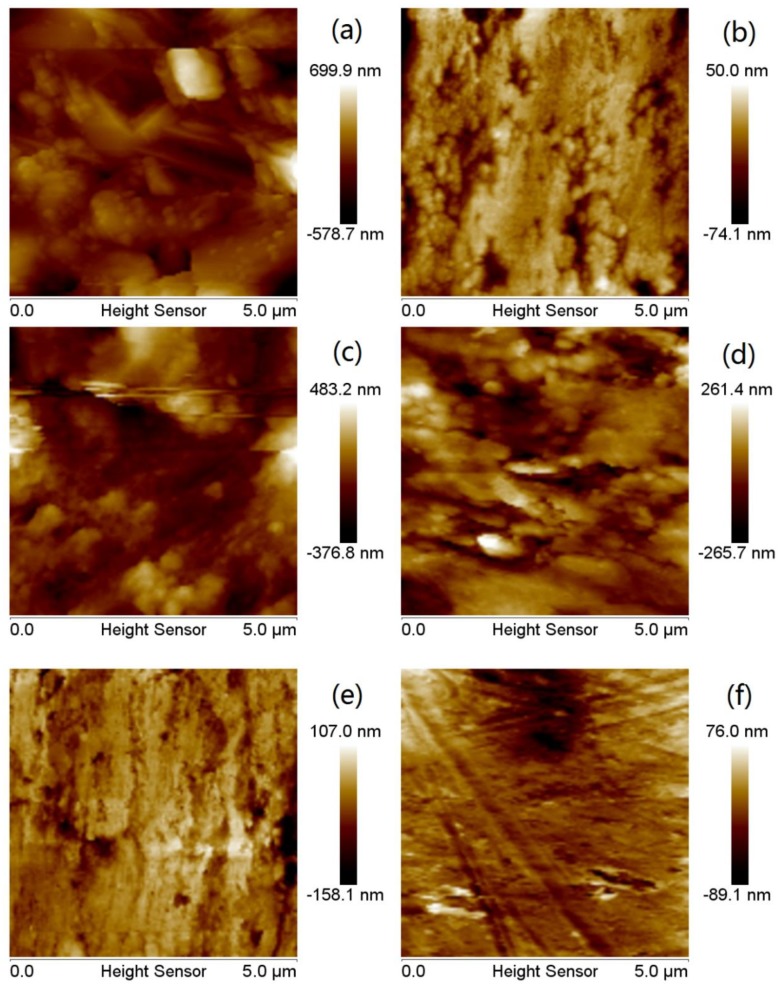
AFM plane topography of N80 steel sheet: (**a**) blank sample; (**b**) initial uncorroded sample; (**c**–**f**) is added 50 mg/L, 100 mg/L, 200 mg/L, and 300 mg/L MBT.

Figure 9a shows the N80 steel sheet without MBT, it can be seen from the figure that the surface corrosion of the N80 steel sheet is serious and the roughness is high because the metal is strongly damaged in the etching solution; Figure 9b is the polished N80 steel sheet that has not been immersed in the etching solution, it can be seen from the figure that the surface of the steel sheet is relatively smooth and the roughness is very low. Based on Figure 9c–f and Figure 10c–f, the N80 steel sheet added to the corrosion inhibitor MBT is significantly different from that of Figure 9a and Figure 10a. As the concentration of the corrosion inhibitor MBT increases, the corrosion rate of the steel sheet reduces, the surface of the steel sheet is smoother and the roughness is also closer to that of Figure 9b. MBT at a concentration of 300 mg/L is exhibited in Figure 9f and Figure 10f with sandpaper polishing marks. It shows that the surface of MBT carbon steel forms an effective protective film, which prevents the corrosion of the steel sheet.

By analysing the data of the atomic force microscope carried by the analysis software (NanoScope Analysis v1.40), we can obtain the mean square roughness (Rq), the average roughness (Ra) and the maximum height difference (Rmax) of the surface of the N80 steel sheet, which is shown in Table 5. It can be seen from the table, that after the N80 blank is etched, the Rq is 155.0 nm, the Ra is 113.0 nm and the Rmax is 2050.0 nm, which is quite different from the Rmax of 165.0 nm which has not been etched. After adding 300 mg/L corrosion inhibitor MBT, the Rmax of the N80 steel sheet is 192.0 nm, which is very close to the maximum height difference of the unetched N80 steel sheet. Combined with Figure 9 and Figure 10, the surface roughness of the steel sheet can be seen. The appearance of the corrosion inhibitor forms a passivation film on the surface of the N80 steel sheet, which reduces the surface roughness of the steel sheet and effectively prevents the N80 steel sheet from being eroded by corrosive media.

## 4. Conclusions

The overall aim of this study was to synthesise and assess the corrosion inhibition effect of MBT. After dosage temperature tests, the corrosion rate was 0.0446 mm/a and the corrosion inhibition rate was 90.4% while the dosage was 400 mg/L in a CO_2_ saturated solution of 3 wt % NaCl, in a 70 °C constant temperature water bath for 72 h. As evidenced by polarisation curve analysis, MBT is a mixed type corrosion inhibitor mainly used for suppressing the cathode. The adsorption of MBT on the metal surface contributes to a dense molecular film, changing the interface properties and electric double layer structure, thus achieving a good corrosion inhibition effect. The adsorption follows the Langmuir isotherm adsorption and the calculated $\Delta G_{\mathrm{ads}}$ < 0 indicates that the chemical adsorption of the corrosion inhibitor is spontaneous on the N80 steel sheet. AFM is applied in microscopic characterisation on the surface. Results demonstrate that the N80 steel sheet added the corrosion inhibitor MBT is different from blank the sample, showing a smooth surface, indicating that MBT contributes to the protective film with a good corrosion inhibition effect on the surface of the steel sheet.

## Figures and Tables

**Table 1:** Experimental factors and levels.

Level	A n(MB-2):n(HCHO):n(CH_3_COCH_3_)	B Temperature/°C	C Time (h)
1	1:1:1	70	4
2	1.5:1.5:1	75	5
3	2:2:1	80	6
4	2.5:2.5:1	85	7

**Table 2:** Orthogonal experimental results.

NO.	A	B	C	Corrosion Rate (mm/a)
1	1	1	1	0.180
2	1	2	2	0.143
3	1	3	3	0.076
4	1	4	4	0.059
5	2	1	2	0.135
6	2	2	1	0.139
7	2	3	4	0.068
8	2	4	3	0.056
9	3	1	3	0.098
10	3	2	4	0.073
11	3	3	1	0.102
12	3	4	2	0.067
13	4	1	4	0.120
14	4	2	3	0.084
15	4	3	2	0.079
16	4	4	1	0.106
I	289.2	259.8	303.2	
II	296.7	302.9	301.0	
III	341.0	338.7	307.8	
IV	308.6	334.2	322.7	
*K*_1_	0.115	0.133	0.132	
*K*_2_	0.100	0.110	0.106	
*K*_3_	0.085	0.081	0.079	
*K*_4_	0.097	0.072	0.080	
*R*	0.03	0.061	0.053	
Effect order	B > C > A			
Optimal level	A_3_	B_4_	C_3_	

**Table 3:** Electrochemical parameters of different MBT concentrations.

Inhibitor	Conc. (mg·L^−1^)	E_corr_ (V)	I_corr_ (μA·cm^−2^)	Ba (mV·dec^−1^)	Bc (mV·dec^−1^)	*η* (%)
MBT	0	−0.725	60.99	10.21	5.11	-
	50	−0.727	26.10	9.86	5.98	57.2
	100	−0.737	12.62	5.59	7.58	79.3
	200	−0.775	10.06	12.06	7.23	83.5
	300	−0.755	7.99	9.74	8.07	86.9

**Table 4:** Electrochemical impedance spectroscopy fitting parameters of different concentrations of MBT.

Inhibitor	Conc. (mg/L)	R_s_ Ω·cm^2^	C_dl_ μF·cm^−2^	R_ct_ Ω·cm^2^	CPE		R_t_ (Ω)	*η*%
					Y_0_ μS·cm^−2^·s^n^	n		
MBT	0	1.579	6.02	136.7	15.33	0.82	13.57	-
	50	4.507	13.96	255	8.83	0.69	23.56	46.4
	100	1.758	7.90	871.7	54.22	0.73	58.71	84.3
	200	1.571	12.13	941.2	53.71	0.67	37.77	85.5
	300	1.867	18.82	1020	10.92	0.61	42.47	86.6

**Table 5:** AFM parameters after soaking in different concentrations of corrosion inhibitor MBT.

MBT (mg/L) + CM	Ra (nm)	Rq (nm)	Rmax (nm)
0 + 0	13.1	17.3	165.0
0 + CM	113.0	155.0	2050.0
50 + CM	93.0	119.0	873.0
100 + CM	58.1	73.2	597.0
200 + CM	24.4	33.2	369.0
300 + CM	15.9	21.6	192.0

Note: CM (corrosive media) refers to a CO_2_ saturated solution containing 3 wt % NaCl.
